# Professional Etiquette

**Published:** 1858-11

**Authors:** 


### *Professional Etiquette.*

The observance of artificial rules in the social intercourse between man and man, has now become universally recognized as one of the necessities of civilized life. The daily routine of existence is but one continued series of formulas, regarded as essential to the character of a gentleman—and a neglect of which involves a forfeiture of social position. Beneath the glossy mask of modern politeness, the savage instincts of the natural man silently lurk, and the ungoverned passions of the greatest of animals, are only held in check by the courtesies and refinements of society. Founded upon the great principles of moral right and justice—taught by conscience and revelation—the rules of politeness, and the observance of good manners are binding on man both in his moral and religious aspect; and all the elaborate details of Chesterfield's Manual are included in the simple but comprehensive language of the *Golden Rule.*

Medical etiquette, as it is usually called by both the profession and the public, founded upon the general principles which govern mankind in the other departments of life, demands from every member of the medical fraternity a conscientious and careful observance.

In the pursuit of an art depending so much on personal qualities—an art imperfect from the necessity of the case, and ever allowing and indeed encouraging differences of opinion and of practice—it is an easy matter to find grounds of dispute and causes of offence. Upon the morbid brain of the invalid, a cast of the eye or a shrug of the shoulder will produce all the effect of the most elaborate insult. The frailties of human nature, exaggerated by disease, are only too prone to seize upon every opportunity to indulge themselves, and hence the common forms of politeness have been drawn more stringently around the profession of medicine—not that its votaries require a stricter rule, but because the temptations to offend are so constantly presented, and so easy of execution. A careful observance of these rules, however irksome and oftentimes, in particular instances, absurd they may appear to be, is the bounden duty of every member of the profession. These things will not make a vulgar man a gentleman, nor will the law stifle rascality, but the yoke which sits easily upon true-hearted and honest men will dreadfully gall the stiff-necked manœuvrer, who longs to escape from its influence. In the struggle to gratify his secret love of intrigue without violating the etiquette of the profession, the claws of the tiger will at last be discovered, and inevitably receive the indignation and frown of his brethren and the public.

We would then earnestly address our young friends more especially, upon the importance of carefully observing all the courtesies of professional etiquette. To the younger member of the faculty, these rules often seem unfair. They appear to act as so many barriers to his advancement in practice. Competition is checked, if not prohibited—the opportunities for making friends and reputations are lost, and the young and ambitious aspirant for medical honors is sorely tempted to spurn these old and conservative customs, whose usefulness he will not acknowledge, and give full rein to his impatient desires for a brilliant and profitable career. If our words should perchance meet the eye of such a restless, and it may be noble brother, let us affectionately point him to the forcible appeal of the brilliant and versatile Holmes, to be found in this number of the Journal; and if one misguided brother, who has foolishly and fatally attempted "to make a law unto himself," is brought back to the path of rectitude and duty, our object will be fully accomplished.— *Virginia Medical Journal.*

